# Inhibition of Euchromatic Histone Methyltransferase 1 and 2 Sensitizes Chronic Myeloid Leukemia Cells to Interferon Treatment

**DOI:** 10.1371/journal.pone.0103915

**Published:** 2014-07-31

**Authors:** Sheng Wei Loh, Wei Lun Ng, Kok Siong Yeo, Yat-Yuen Lim, Chee-Kwee Ea

**Affiliations:** Institute of Biological Sciences, Faculty of Science, University of Malaya, Kuala Lumpur, Malaysia; Ludwig-Maximilians-Universität München, Germany

## Abstract

**Background:**

H3K9 methylation is one of the essential histone post-translational modifications for heterochromatin formation and transcriptional repression. Recently, several studies have demonstrated that H3K9 methylation negatively regulates the type I interferon response.

**Results:**

We report the application of EHMT1 and EHMT2 specific chemical inhibitors to sensitize CML cell lines to interferon and imatinib treatments. Inhibition of EHMT1 and EHMT2 with BIX01294 enhances the cytotoxicity of IFNα2a in four CML cell lines, K562, KCL22, BV173 and KT1 cells. Chromatin immunoprecipitation assay shows that BIX01294 treatment enhances type I interferon response by reducing H3K9me2 at the promoters of interferon-stimulated genes. Additionally, BIX01294 treatment augments IFNα2a- and imatinib-mediated apoptosis in CML cell lines. Moreover, our data suggest that the expression level of EHMT1 and EHMT2 inversely correlates with the type I interferon responsiveness in CML cell lines.

**Conclusions:**

Our study sheds light on the role of EHMT1 and EHMT2 as potential targets in improving the efficacy of standard treatments of CML.

## Introduction

Type I interferons (IFN), including IFNα, IFNβ and IFNδ are secreted glycoproteins with anti-proliferative, antiviral and immunoregulatory properties. Type I interferons bind to IFNAR1 and IFNAR2, and regulate gene expression through JAK/STAT pathway [1]. Among the type I interferons, IFNα is an important therapeutic cytokine that exerts antitumor activity in a variety of tumor cells.

Chronic myeloid leukemia (CML) is one of the hematologic malignancies that responses well to IFN-α therapy. CML is characterized by the presence of Philadelphia chromosome. The molecular pathogenesis of CML arises from the consequences of the Philadelphia chromosome formation [2]. The Philadelphia chromosome results from chromosomal translocation between the *ABL* gene on chromosome 9 and the *BCR* gene on chromosome 22 to form the fusion *BCR-ABL* gene. *BCR-ABL* encodes a constitutively active tyrosine kinase. IFNα suppresses the proliferation of Philadelphia-positive CML cells, and induces both hematologic and cytogenetic remission with the disappearance of Philadelphia clones [3].

Recently, several studies showed that interferon-stimulated genes (ISGs) are negatively regulated by the H3K9 methylation [4], [5]. Two histone methyltransferases, euchromatic histone methyltransferase 1 and 2 (EHMT1 and EHMT2; also known as GLP and G9a), play an essential role in regulating the type I interferon response [4], [5]. Inhibition of EHMT2 by gene knockout in mice or inhibition of EHMT1 and EHMT2 with a chemical inhibitor, BIX01294 [6], enhances type I interferon response and protect cells from viral infection.

In this study, we demonstrate that inhibition of EHMT1 and EHMT2 with specific chemical inhibitors in several CML cell lines sensitizes cells to interferon and imatinib treatments. We further show that inhibition of EHMT1 and EHMT2 in CML cells enhances interferon-induced expression of ISGs and apoptosis. We describe a reverse correlation between the expression levels of EHMT1 and EHMT2 and the sensitivity of CML cell lines to interferon treatment and VSV infection.

## Materials and Methods

### Cell Culture

HeLa (ATCC) and HaCat (ATCC) cells were cultured in DMEM supplemented with 10% fetal bovine serum (FBS), penicillin G (100 U/ml), and streptomycin (100 µg/ml). K562 (ATCC), KCL22 [7], BV173 (DSMZ), KT1 [8] and Jurkat (ATCC) cells were maintained in RPMI supplemented with 10% FBS, penicillin G (100 U/ml), and streptomycin (100 µg/ml).

### Antibodies and compounds

Antibodies against PARP1 (F2), histone H3 (C16), actin (I-19) and Hsp90 (C20) were purchased from Santa Cruz Biotech. Antibodies against BCR-Abl (Cell Signaling), H3K9me2 (Abcam, ab1220), cleaved caspase-3 (Cell Signaling), EHMT2 (EMD Millipore) and EHMT1 (R&D systems) were purchased from the respective commercial sources. BIX01294 and UNC0638 were purchased from Sigma-Aldrich.

### Cell proliferation assay

Cells were treated with or without various concentration of BIX01294 together with or without various concentration of IFNα2a in a 96 wells format. After incubation for four days, 10 µl of 2 mg/ml 3-(4, 5-dimethylthiazol-2-yl)-2, 5-diphenyltetrazolium bromide (MTT) in DMEM medium was added and cells were further incubated for three hours at 37°C in a CO_2_ incubator. Cells were spun down at 2500 rpm for 5 minutes and the medium was carefully removed. One hundred and fifty microliter of DMSO was added to each well. After pipetting up and down several times, the absorbance was measured with a M200 PRO microplate reader (Tecan) at the wavelength of 540 nm.

### Stable shRNA transduction

ShRNA plasmids against human EHMT1 (sc-62261-SH), human EHMT2 (sc-43777-SH) and empty vector tet-pLKO-puro (addgene) were purchased from the respective sources, and lenti-viruses were produced according to the manufacturer’s protocol. K562 cells were infected with lenti-viruses carrying control, EHMT1 shRNA or EHMT2 shRNA. After 24 hours, culture media were removed and replaced with fresh media supplemented with 1 µg/ml puromycin. The cells were selected with puromycin for two weeks.

### Ectopic expression of mEHMT1 and mEHMT2

PMSCV-FLAG-mEHMT1 and pCDNA3-HA-mEHMT2 plasmids were co-transfected into KT1 cells using lipofectamine 2000 according to manufacturer’s protocol. Forty-eight hours post-transfection, the cells were selected with 1 µg/ml puromycin and 1.2 mg/ml G418 for two weeks.

### RT-PCR

Cells pretreated with or without BIX01294 or UNC0638 for 24 hours were incubated with IFNα2a for two hours. Total RNAs were isolated with the Thermo Scientific GeneJET RNA Purification Kit. Complementary DNAs were synthesized and Quantitative PCR was performed with 2X SYBR Green PCR Master mix (Thermo Scientific) and run on a Bio-Rad CFX 96 Real-Time PCR System. All data were then normalized to *L32*. The sequences of the primers are listed in table S1.

### ChIP-qPCR

ChIP assays using 2×10^6^ cells per reaction were performed as recently described [9] with minor modification. Antibodies included anti-histone H3 (ab1791; Abcam), and anti-dimethyl histone H3K9 (ab1220; Abcam), anti-RNA Polymerase II (Cat. 05-623B; UpState) and mouse normal IgG (I5381; Sigma) were purchased from respective sources. Briefly, 1.2×10^7^ cells were cross-linked in 1% formaldehyde for 10 minutes at room temperature with gentle rocking or inversion every 2–3 minutes. Cells were pelleted by centrifugation (300 g for 5 minutes), and washed twice in ice-cold 1x PBS. The cells were lysed in 300 µl of lysis buffer (10 mM Tris pH 7.5, 1 mM EDTA, 1% SDS) containing protease inhibitor cocktail (PIC; Sigma) and incubated on ice for 10 minutes. After lysis, 900 µl of 1x PBS containing PIC was added and 300 µl was aliquoted into individual 1.5 ml tubes. Each 300 µl aliquot was sonicated by using a bioruptor plus sonicator (Diagenode), which was empirically determined to give rise to genomic fragments ∼200–500 bp. The soluble chromatin was collected by 4°C ultracentrifugation (13,000 rpm for 10 minutes) and pooled into a new 15 ml falcon tube. The supernatant was diluted 2-fold with 2x RIPA buffer (10 mM Tris-HCl pH 7.5, 1 mM EDTA, 1% Triton X-100, 0.1% SDS, 0.1% sodium deoxycholate, 100 mM NaCl, PIC), 1/10 volume (40 µl) input was removed, and 400 µl of soluble chromatin (equivalent to 2×10^6^ cells) was distributed to new 1.5 ml tubes. Each respective antibody was added at appropriate amount as tested in titration experiments using control promoters. Immunoprecipitations (IPs) were performed overnight at 4°C with rotation, and antibody: protein:DNA complexes were then collected with 15 µl of protein A and G magnetic beads (Thermo Fisher) for 4 hours of rotation. The beads were washed three times using 200 µl of RIPA buffer and once with TE (10 mM Tris-HCl pH 8.0, 10 mM EDTA) buffer, then incubated with 200 µl of fresh elution buffer (50 mM Tris-HCl pH 7.5, 5 mM EDTA, 1% SDS, 50 mM NaCl) with proteinase K for 2 hours in a thermomixer (1300 rpm, 68°C) to reverse the protein:DNA cross-links. After incubation, eluates were collected into new 1.5 ml tubes. Genomic DNA was recovered by using phenol chloroform extraction and ethanol precipitation. Pellets were washed in 70% ethanol, briefly air-dried, and resuspended in TE buffer. Quantitation of ChIP DNA (relative enrichment) was performed using a CFX Connect Real-Time PCR Detection System (Bio-Rad) with ABI SYBR select master mix(Life Technologies) and ChIP qPCR primer sequences as listed in Table S2. Enrichment of histone modifications at genomic regions were expressed as % input. % Input was calculated using the formula % (ChIP/Total input) = 2∧[(Ct(x% input)–log(x%)/log2)–Ct(ChIP)]×100% to account for chromatin sample preparation differences. Ct (ChIP) and Ct (x% input) are threshold values obtained from exponential phase of qPCR for the IP’d DNA sample and input sample respectively; the compensatory factor (log(x%)/log2) is used to take into account the dilution 1:x of the input.

### Flow Cytometry

For cell cycle analysis, K562 cells were treated with IFNα2a or imatinib in the presence or absence of BIX01294 for two days. Cells were washed with PBS and fixed with 70% ethanol. Fixed cells were stained with PI and analyzed using a MACSQuant Analyzer (Miltenyi Biotec). For viral infection, K562, KCL22, BV173 and KT1 cells were infected with or without VSV-GFP at MOI of 0.5 for 24 hours. Cells were fixed with 0.1% formaldehyde and analyzed using the MACSQuant Analyzer. Data were further analyzed with FlowJo software.

### Statistical Analysis

Data were analyzed by Microsoft Excel and presented as the mean±SD. Data are representative of three or more independent experiments.

## Results

### Inhibition of EHMT1 and EHMT2 with BIX01294 enhances type I interferon response in K562 cells

**Figure 1:**
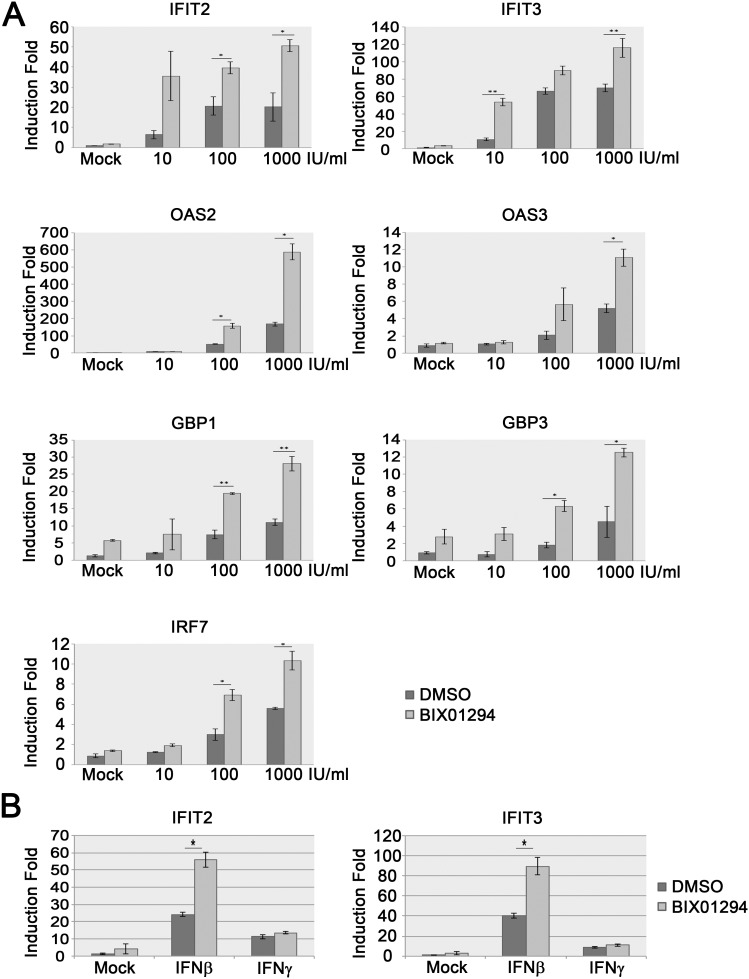
BIX01294 enhances the expressions of ISGs in K562 cells. (**A**) K562 cells were incubated with 2.5 µM BIX01294 for 24 hours. The cells were then treated with various concentrations of IFNα2a as indicated. After two hours of IFNα2a stimulation, the expression of various ISGs was measured with RT-qPCR. Error bars represent the variation range of duplicate experiments. *: p<0.05, **: p<0.01. (**B**) K562 cells were incubated with 2.5 µM BIX01294 for 24 hours. The cells were then treated with IFNβ or IFNγ for two hours. The expression of *IFIT2* and *IFIT3* was measured with RT-qPCR. Error bars represent the variation range of duplicate experiments. *: p<0.05.

We previously showed that inhibition of EHMT1 either with RNA interference (RNAi) or a chemical inhibitor enhances interferon response and protects cells from virus infection [4]. To test if inhibition of EHMT1 and EHMT2 in CML cells enhances their response to interferon, we treated K562 cells, a CML cell line, with an EHMT1 and EHMT2 specific inhibitor, BIX01294 [6], [10], and measured the expression of ISGs with RT-qPCR. Treating K562 cells with BIX01294 for twenty-four hours led to an 83% reduction of the global level of H3K9me2 (Figure S1A) confirming the inhibition of EHMT1 and EHMT2 by BIX01294 in K562 cells. Importantly, pretreating K562 cells with BIX01294 significantly enhanced IFNα2a-induced gene expression (Figure 1A). We treated K562 cells with an increasing dosage of IFNα2a and observed a dose dependent induction of several ISGs, including *IFIT2, IFIT3, GBP1, GBP3, OAS2, OAS3* and *IRF7*. In the presence of BIX01294, 100 IU/ml of IFNα2a produced a level of all tested ISGs similar to that produced by 1000 IU/ml IFNα2a-treated DMSO-treated control K562 cells. Moreover, we showed that the protein level of IRF7 increased in response to IFNα2a stimulation (Figure S1B). Pretreating K562 cells with BIX01294 increase the protein level of IRF7 even in the absence of IFNα2a stimulation.

To test if BIX01294 treatment enhances the expression of ISGs induced by other interferons in K562 cells, we pretreated K562 cells with or without BIX01294 followed by IFNβ (a type I interferon) or IFNγ (a type II interferon) stimulation. Consistent with IFNα2a stimulation, pretreating K562 cells with BIX01294 enhanced IFNβ-induced, but not IFNγ-induced expression of *IFIT2* and *IFIT3* (Figure 1B). These results indicate that inhibition of EHMT1 and EHMT2 in K562 cells significantly enhances the expression of ISGs in response to type I interferons but not type II interferon stimulation.

### BIX01294 sensitizes CML cell lines to IFNα2a treatment

**Figure 2:**
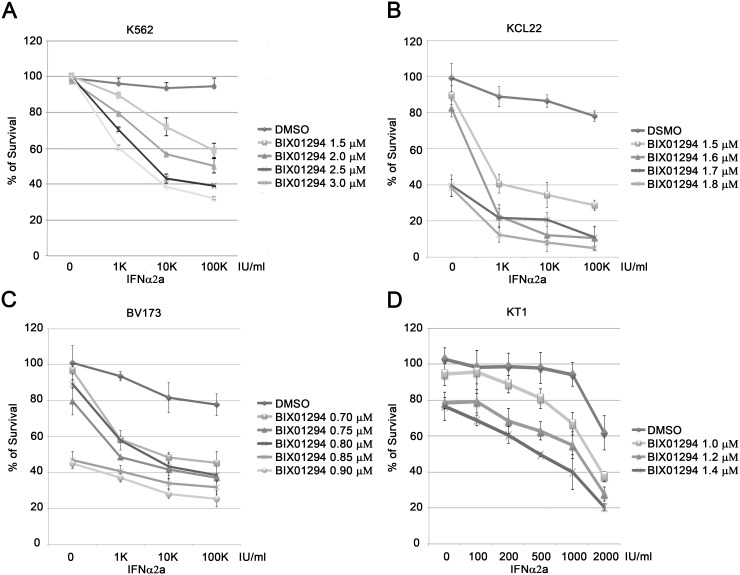
BIX01294 inhibits the proliferation of CML cells. K562 (**A**), KCL22 (**B**), BV173 (**C**) and KT1 (**D**) cells were cultured with various concentrations of BIX01294 and IFNα2a as indicated. After four days, cell proliferation was measured with a MTT assay. Results represent the mean ± SD in quadruplicate experiments.

IFNα2a is approved by the US FDA as an anticancer drug against CML [11]. K562 cells have been shown to be resistant to interferon treatment *in vitro* (IC_50_>10^5^ IU/ml) [12], [13]. To test whether inhibition of EHMT1 and EHMT2 renders K562 cells sensitive to interferon treatment, we measured the proliferation of K562 cells treated with or without IFNα2a in the presence or absence of BIX01294, using a MTT assay. Consistent with previous reports [12], [13], we found that K562 cells were resistant to IFNα2a treatment because treatment with 10^5^ IU/ml of IFNα2a only reduced cell proliferation by 10% (Figure 2A). Interestingly, in the presence of BIX01294, IFNα2a strongly inhibited the proliferation of K562 cells. For example, in the presence of 3 µM of BIX01294, 1000 IU/ml of IFNα2a achieved 40% inhibition of cell proliferation and 10^5^ IU/ml of IFNα2a inhibited cell proliferation by 60% (Figure 2A). Moreover, treating K562 cells with 3 µM BIX01294 alone had no effect on their proliferation but a higher dose was toxic (data not shown). Our findings demonstrate that inhibition of EHMT1 and EHMT2 sensitizes K562 cells at least by a 100-fold to interferon treatment.

**Figure 3:**
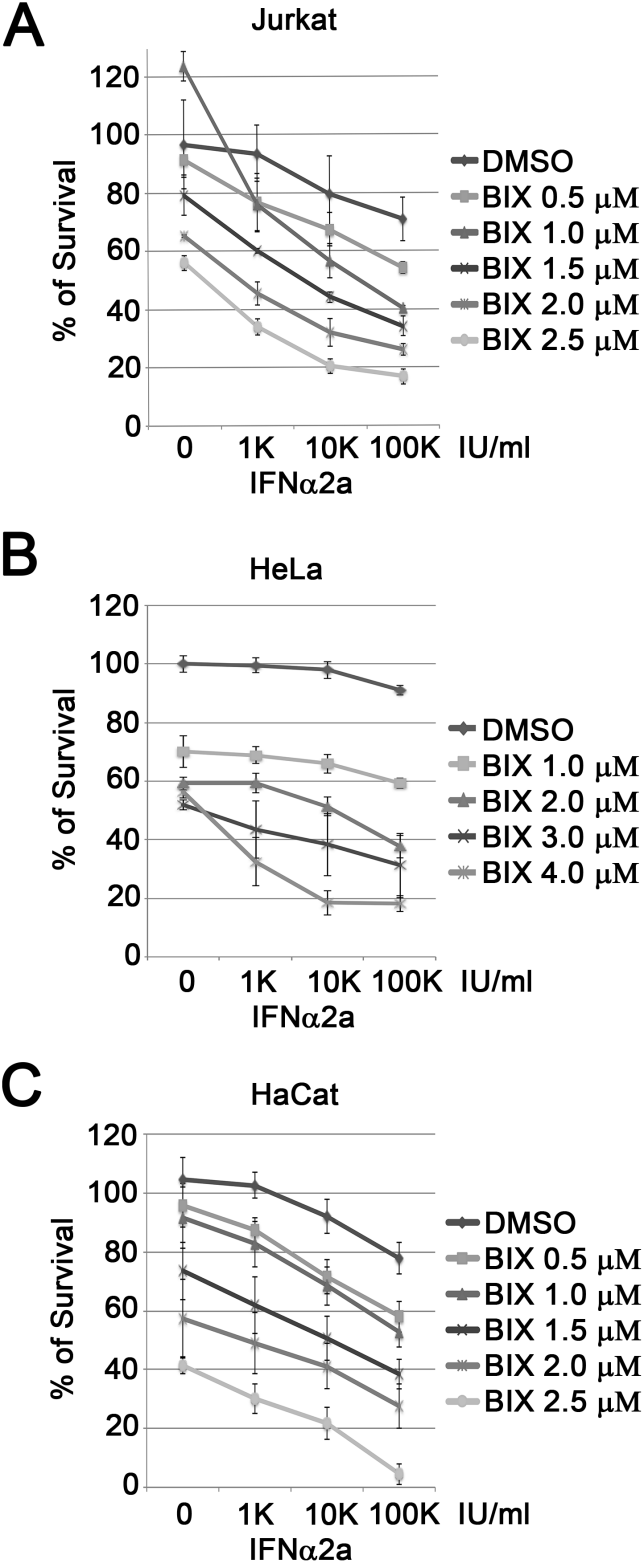
BIX01294 slightly enhance IFNα2a-induced anti-proliferation in non-CML cells. Jurkat (**A**), HeLa (**B**) and HaCat (**C**) cells were cultured with various concentrations of BIX01294 and IFNα2a as indicated. After four days, cell proliferation was measured with a MTT assay. Results represent the mean ± SD in quadruplicate experiments.

To make sure that the phenomenon we observed is not unique to K562 cells, we tested three additional CML cell lines, KCL22, BV173 and KT1. Among these cell lines, KT1 cells were sensitive to IFNα2a, while KCL22 and BV173 cells were resistant to IFNα2a (Figure 2B–D). Consistently, BIX01294 treatment significantly sensitized KCL22 and BV173 cells to IFNα2a treatment. Furthermore, inhibition of EHMT1 and EHMT2 with BIX01294 moderately enhanced the cytotoxicity of IFNα2a in interferon-sensitive KT1 cells. In addition to CML cell lines, we also tested three additional cell lines with different origins, Jurkat (a T cell lymphoma), HeLa (a cervical cancer cell), and HaCat (an immortal human keratinocyte). None of these cells proved sensitive to IFNα2a treatment as only about 20% inhibition of cell proliferation was observed at the highest concentration of IFNα2a tested (10^5^ IU/ml) (Figure 3A–C). Unlike responses observed in CML cell lines, inhibition of EHMT1 and EHMT2 in Jurkat, HeLa and HaCat cells had moderate or little effect on the sensitivity of these cells to interferon treatment. Taken together, these results suggest that inhibition of EHMT1 and EHMT2 with BIX01294 sensitizes CML cells, and to a lesser extent on Jurkat, HeLa or HaCat cells to interferon treatment.

### Inhibition of EHMT1 and EHMT2 with UNC0638 enhances interferon response in K562 cells

**Figure 4:**
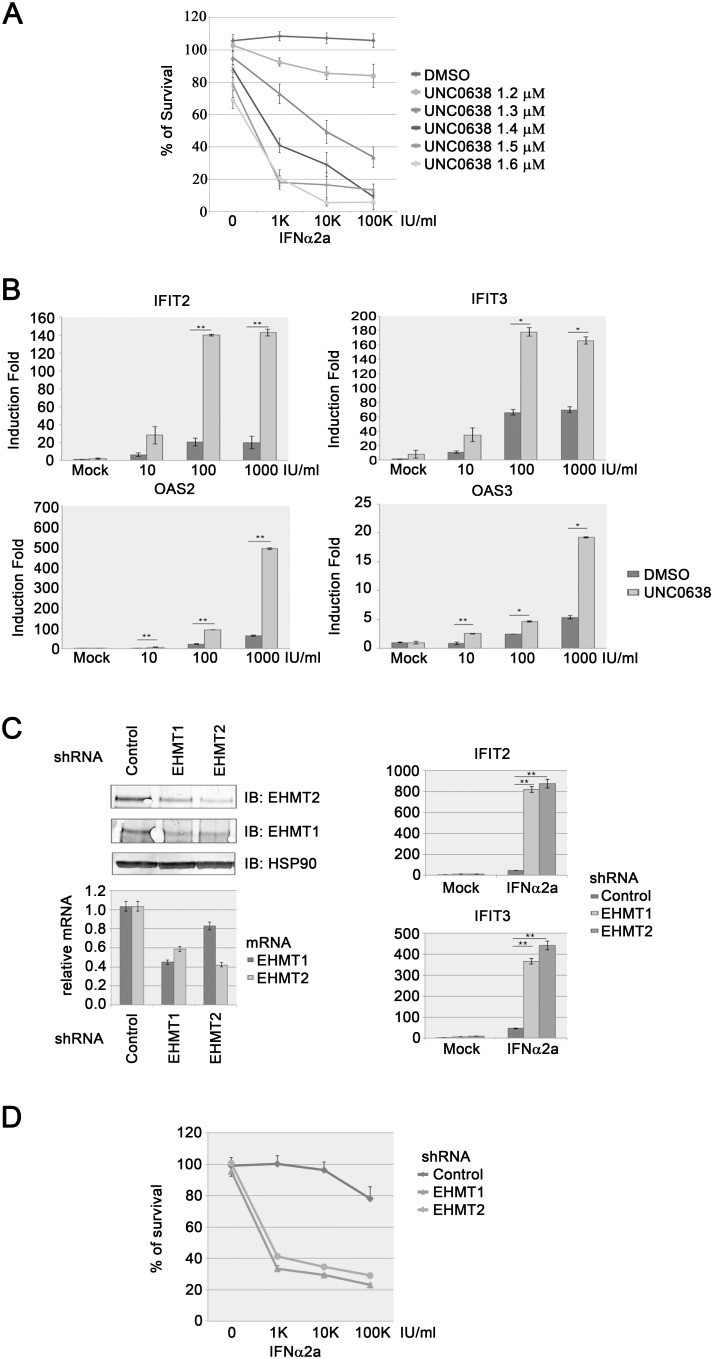
UNC06398 inhibits the proliferation of K562 cells and potentiates the expression of ISGs. (**A**) K562 cells were cultured with various concentrations of UNC0638 and IFNα2a as indicated. After four days, cell proliferation was measured with a MTT assay. Results represent the mean ± SD in quadruplicate experiments. (**B**) K562 cells were incubated with 5 µM UNC0638 for 24 hours followed with various concentrations of IFNα2a stimulation as indicated. After two hours of IFNα2a stimulation, the expression of various ISGs was measured with RT-qPCR. Error bars represent the variation range of duplicate experiments. *: p<0.05, **: p<0.01. (**C**) Whole cell extracts or total RNA were generated from K562 cells infected with control or lentiviruses carrying EHMT1- or EHMT2-specific shRNAs (left). EHMT1 or EHMT2 protein levels were analyzed by immunoblotting using indicated antibodies while mRNA levels were measured with RT-qPCR. Error bars represent the variation range of duplicate experiments. The same cells were stimulated with 1000 IU/ml IFNα2a for two hours (right). The expression of various ISGs was measured with RT-qPCR**.** Error bars represent the variation range of duplicate experiments. **: p<0.01. (**D**) K562 cells as in (**C**) were cultured with various concentrations of IFNα2a as indicated. After four days, cell proliferation was measured with a MTT assay. Results represent the mean ± SD in quadruplicate experiments.

To minimize the possibility that the effect we observed with BIX01294 treatment was caused by an off-target effect, we tested a second commercially available EHMT1- and EHMT2-specific inhibitor, UNC0638 [10]. Treating K562 cells with UNC0638 for twenty-four hours reduced the global level of H3K9me2 by 79%, which was comparable to BIX01294 (Figure S1). Next, we tested the effect of UNC0638 on IFNα2a-mediated anti-proliferation. Similar to BIX01294 treatment, inhibition of EHMT1 with UNC0638 sensitized K562 cells to interferon treatment (Figure 4A). Furthermore, we measured interferon response with RT-qPCR measurement showed that treating K562 cells with UNC0638 enhanced the expression of several ISGs in response to IFNα2a stimulation, including *IFIT2, IFIT3, OAS2,* and *OAS3* (Figure 4B).

### Knocking down expression of either EHMT1 or EHMT2 with RNAi enhances interferon response in K562 cells

In addition, we knocked down the expression of either EHMT1 or EHMT2 with stablely transduced short hairpin RNAs (shRNAs) against EHMT1 or EHMT2 in K562 cells (Figure 4C). Infecting K562 cells with lenti-viruses carrying shRNAs against EHMT1 or EHMT2 achieved a 50–60% knockdown of the mRNA as well as the protein levels of EHMT1 and EHMT2 (Figure 4C, left panel). It has been previously shown that EHMT1 and EHMT2 form functional heterodimer and knocked out of either EHMT1 or EHMT2 destabilizes EHMT2 or EHMT1 respectively [5], [14]. Similar phenomena were observed in our shRNAs-transduced K562 cells. Consistently, we showed that IFNα2a-induced expression of *IFIT2* and *IFIT3* were dramatically enhanced with the knocked down of either EHMT1 or EHMT2 (Figure 4C, right panel). Moreover, knocked down expression of either EHMT1 or EHMT2 sensitized K562 cells to interferon treatment (Figure 4D). Taking together, our results show that two different EHMT1- and EHMT2-specific chemical inhibitors, as well as knocking down the expression of either EHMT1 or EHMT2 enhance interferon response in CML cell lines, implying that inhibition of EHMT1 and EHMT2 sensitizes interferon resistant CML cell lines to interferon-mediated anti-proliferation.

### EHMT1 and EHMT2 catalyze H3K9 methylation at the promoters of ISGs

**Figure 5:**
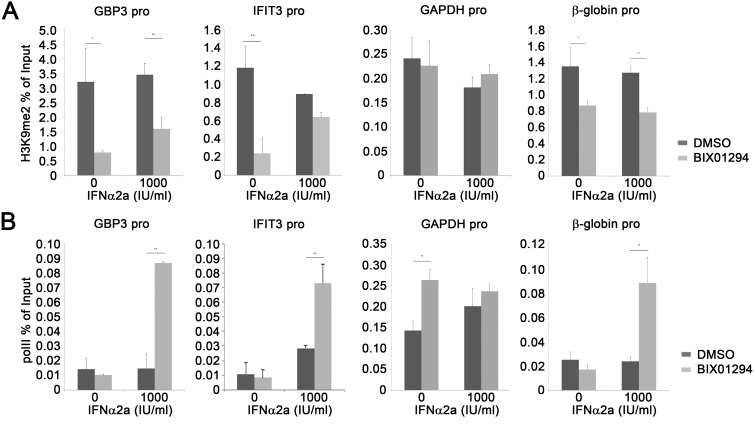
EHMT1 and EHMT2 promote H3K9 methylation at the promoters of *GBP3* and *IFIT3*. (**A–B**) K562 cells treated with DMSO or BIX0129 were stimulated with IFNα2a for 2 hours, and were analyzed by ChIP (H3K9me2 (**A**) and polII (**B**); mean±SD; *: p<0.05;**: p<0.01; representative data of three independent experiment).

To determine if EHMT1 and EHMT2 catalyze the H3K9 methylation at the promoters of ISGs, we analyzed the levels of H3K9me2 in DMSO- or BIX01294-treated K562 cells stimulated with or without IFNα2a. In BIX01294-treated K562 cells, the basal levels of H3K9me2 were reduced by 75–80% at the *GBP3* and *IFIT3* promoters, and 36% at the *β*-*globin* promoter (Fig. 5A). Accordingly, low level of H3K9me2 was detected at the promoter of highly expressed house keeping gene, *GAPDH* and no reduction of the basal level of H3K9me2 at the promoter of *GAPDH* was observed in BIX01294-treated K562 cells. Furthermore, IFNα2a induced more polII recruitment to the promoters of *GBP3* and *IFIT3* in BIX01294-treated K562 cells than in DMSO-treated K562 cells (Figure 5B). This is consistent with the observation that more *GBP3* and *IFIT3* were produced in BIX01294-treated K562 cells in response to IFNα2a stimulation (Figure 1A). These results suggest that EHMT1 and EHMT2 are the major H3K9 HMT that catalyzes H3K9me2 at the *GBP3* and *IFIT3* genes.

### Inhibition of EHMT1 and EHMT2 with BIX01294 enhances cytotoxicity of imatinib in K562 cells

**Figure 6:**
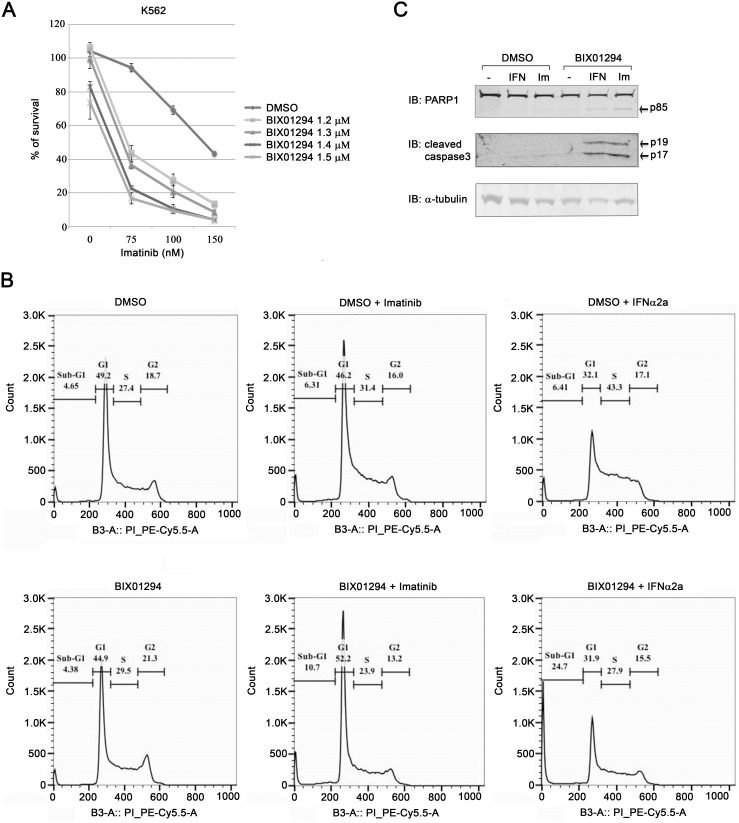
BIX01294 enhances imatinib- and IFNα2a-induced apoptosis in K562 cells. (**A**) K562 cells were cultured with various concentrations of BIX01294 and imatinib as indicated. After four days, cell proliferation was measured with a MTT assay. Results represent the mean ± SD in quadruplicate experiments. (**B**) K562 cells were treated with or without IFNα2a (15 k IU/ml), or imatinib (150 nM) in the presence or absence of BIX01294 (2 µM) for 2 days. Cells were washed with PBS and fixed with 70% ethanol. Fixed cells were then stained with PI and analyzed with FACS. (**C**) K562 cells were stimulated with or without IFNα2a (10 k IU/ml), or imatinib (75 nM) in the presence or absence of BIX01294 (2 µM) for 2 days. Whole cell extracts were prepared and subjected to immunoblotting using the indicated antibodies.

CML is a unique disease, universally characterized by the presence of *BCR-ABL* fusion genes, which encodes a constitutively active tyrosine kinase, and is considered responsible for the pathogenesis of CML [15]. Imatinib (Gleevec) is the first BCR-ABL specific tyrosine kinase inhibitor approved by the FDA for treating CML [16]. To test if inhibition of EHMT1 and EHMT2 enhances the anticancer effect of imatinib in CML, we investigated the cytotoxicity of imatinib in the presence or absence of BIX01294. We demonstrated that K562 cells were very sensitive to imatinib treatment in that 150 nM of imatinib reduced the proliferation of K562 cells by 57% (Figure 6A). More importantly, treating K562 cells with imatinib together with BIX01294 significantly enhanced the anti-proliferation effect of imatinib (Figure 6A). Additionally, imatinib treatment reduced the mRNA and protein level of BCR-ABL while BIX01294 or IFNα2a treatment did not (Figure S2a and b). However, treating K562 cells together with BIX01294 and imatinib did not further reduce the mRNA and protein level of BCR-ABL. These results suggest that inhibition of EHMT1 and EHMT2 sensitizes CML cells to imatinib treatment.

### BIX01294 enhances IFNα2a- and imatinib-induced apoptosis

To investigate if BIX01294 enhances the anti-proliferation effect of IFNα2a and imatinib by inducing cell cycle arrest or apoptosis, we performed cell cycle analysis of K562 cells treated with IFNα2a or imatinib in the presence or absence of BIX01294 with FACS. Treating K562 cells with IFNα2a induced S phase arrest, while imatinib treatment slightly increased the percentage of cells in the sub-G1 phase (Figure 6B). In the presence of BIX01294, cells in the sub-G1 phase were significantly increased after prolonged IFNα2a or imatinib treatments. The increased of cell death was not caused by BIX01294 itself, as treating K562 cells with BIX01294 alone did not increase in the number of sub-G1 cells. However BIX01294 treatment did lead to a slight increase in the number of G2 cells (Figure 6B). These results suggest that inhibition of EHMT1 and EHMT2 enhances IFNα2a- and imatinib-induced cell death. To test if BIX01294 enhances IFNα2a- and imatinib-induced cell death through apoptosis, we monitored the cleavage of the nuclear caspase substrate poly-ADP-ribose polymerase (PARP) and procaspase-3, two biochemical markers for apoptosis. Under our experimental conditions, DMSO-treated K562 cells did not undergo apoptosis in response to IFNα2a or imatinib treatments as only the full length PARP and no cleaved caspase-3 were detected (Figure 6C). On the other hand, combination of BIX01294 with IFNα2a- or imatinib-induced apoptosis in K562 cells as indicated by the generation of a shorter form of PARP (p85) and activated caspase-3 (p17 and p19) (Figure 6C). Thus, inhibiting EHMT1 and EHMT2 with BIX01294 sensitizes K562 cells to IFNα2a- and imatinib-induced apoptosis.

### CML cell lines show different amplitude of interferon response

**Figure 7:**
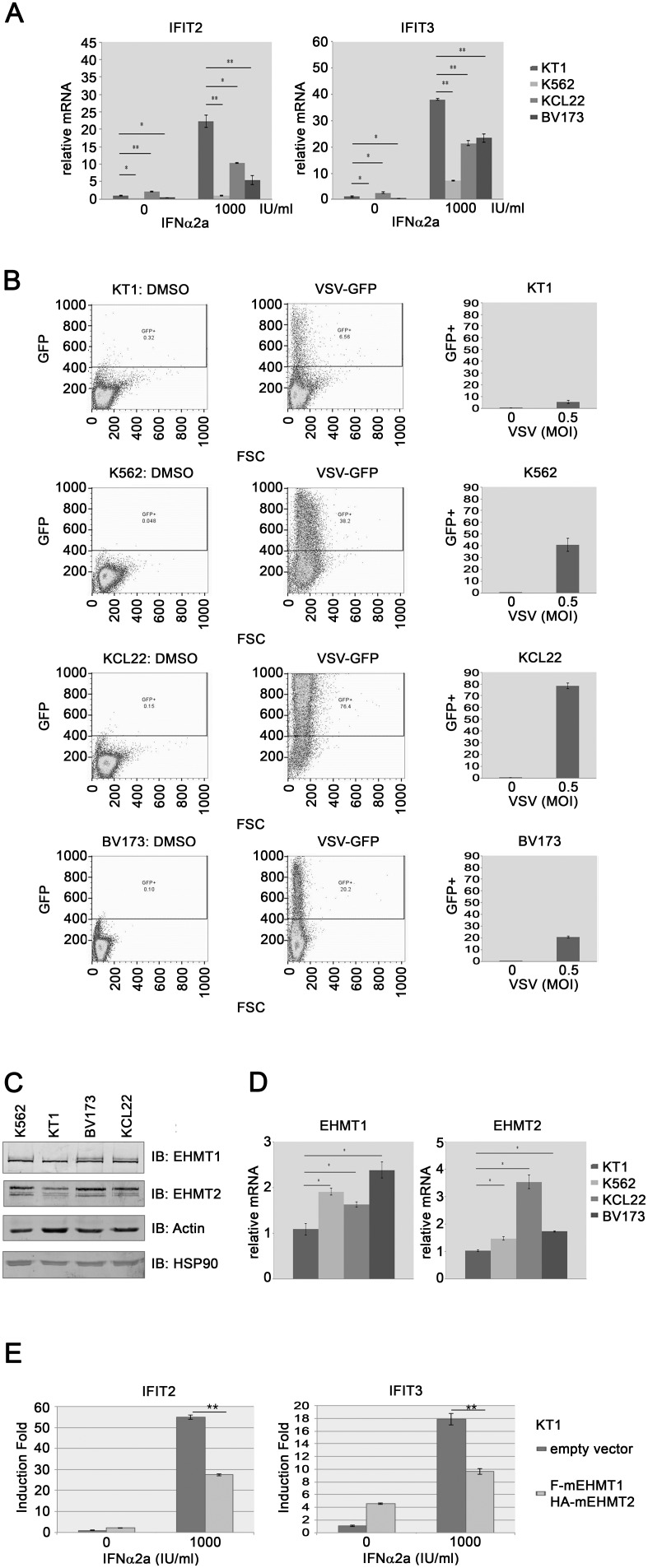
Expression level of EHMT1 inversely correlates with the sensitivity of CML cells to interferon. (**A**) KT1, K562, KCL22 and BV173 cells were treated with or without 1000 IU/ml IFNα2a for 2 hours, the expression of *IFIT2* and *IFIT3* was measured with RT-qPCR. Error bars represent the variation range of duplicate experiments. *: p<0.05, **: p<0.01. (**B**) KT1, K562, KCL22 and BV173 cells were incubated with or without 2.5 µM BIX01294 for 24 hours. Cells were then infected with VSV-GFP at a MOI of 0.5 for 24 hours. GFP positive cells were sorted by FACS. Results represent the mean ± SD in triplicate experiments (**C)** Whole cell extracts were prepared from K562, KT1, BV173 and KCL22 cells, and examined by immunoblotting using the indicated antibodies. (**D**) The relative mRNA levels of EHMT1 and EHMT2 were measured with RT-qPCR. Results represent the mean ± SD in quadruplicate experiments. *: p<0.05. (**E**) Empty vector or FLAG-mEHMT1-HA-mEHMT2 KT1 cells were treated with or without IFNα2a (1000 IU/ml) for two hours, the expression of *IFIT2* and *IFIT3* was measured with RT-qPCR**.** Error bars represent the variation range of duplicate experiments. **: p<0.01.

We observed that K562, KT1, BV173 and KCL22 cells show different sensitivity to IFNα2a (Figure 2). To test if IFNα2a induces different expression levels of ISGs among K562, KT1, BV173 and KCL22 cells, we treated K562, KT1, BV173 and KCL22 cells with or without 1000 IU/ml IFNα2a and measured the expression of ISGs by RT-qPCR. We found that KT1 cells expressed higher basal and induced levels of IFIT2 and IFIT3 relative to K562, BV173 and KCL22 cells (Figure 7A). Since type I interferons are potent antiviral agents, we reasoned that K562, KT1, BV173 and KCL22 cells may have different sensitivity to viral infection. To investigate if KT1 cells are more resistant to viral infection, we infected K562, KT1, BV173 and KCL22 cells with vesicular stomatitis virus carrying a GFP reporter (VSV-GFP) at a multiplicity of infection (MOI) of 0.5. After twenty-four hours, infected cells were quantified by sorting the virus-infected, GFP positive cells using FACS. Consistent with the higher interferon response observed in KT1 cells, KT1 cells were more resistant to VSV-GFP infection compared to K562, BV173 and KCL22 cells (Figure 7B). At the MOI of 0.5, 40.9% of K562 cells, 20.8% BV173 cells and 78.3% KCL22 cells expressed GFP, while only 5.5% of KT1 cells were GFP positive at twenty-four hours post-infection.

It has been previously shown that the expression level of EHMT1 and EHMT2 inversely correlates with the magnitude of type I interferon response [5]. To test if the expression level of EHMT1 and EHMT2 contributes to different expression level of ISGs among KT1, BV173, KCL22 and K562 cells, we compared the protein levels of several proteins among KT1, BV173, KCL22 and K562 cells (Figure 7C). We found that K562, BV173 and KCL22 cells expressed EHMT1 and EHMT2 at a slightly higher level compared to KT1 cells. Furthermore, we also measured the relative mRNA level of EHMT1 and EHMT2 in BV173, KCL22, K562 and KT1 cells with RT-qPCR. We demonstrated that KT1 cells expressed less mRNA of EHMT1 and EHMT2 than BV173, KCL22 and K562 cells (Figure 7D). To further test if the low expression of EHMT1 and EHMT2 in KT1 cells attributes to higher ISGs expression in KT1 cells in response to interferon stimulation, we overexpressed FLAG-mEHMT1 and HA-mEHMT2 in KT1 cells and measured the interferon response by RT-qPCR. The expression of exogenous mouse EHMT1 and EHMT2 were verified by immunoblotting with FLAG-specific and HA-specific antibodies and RT-qPCR (Figure S3). Indeed we found that increase the expression of EHMT1 and EHMT2 significantly reduced the IFNα2a-induced expression of *IFIT2* and *IFIT3* (Figure 7E). Taken together, these results imply that intrinsic expression level of EHMT1 and EHMT2 inversely correlates with the type I interferon responsiveness of CML cells.

## Discussion

We have found that inhibiting the H3K9me2 specific methyltransferases, EHMT1 and EHMT2, either with chemical inhibitors or RNAi sensitizes CML cell lines to interferon treatment. EHMT1 and EHMT2 negatively regulate type I interferon response by promoting H3K9me2 at the promoters of ISGs. We further showed that the expression level of EHMT1 and EHMT2 inversely correlates with the type I interferon response in CML cells.

Treatment strategy of CML has changed from chemotherapy to interferon, and finally to tyrosine kinase inhibitors such as imatinib. Large-scale clinical trials have proven imatinib has high and persistent efficacy in treating CML [17]. However, 25% of patients with imatinib monotherapy show primary refractory disease and drug resistance [18]. Several clinical studies have shown some advantages of the combination of imatinib and IFNα [19]. One of the rationales of the combination therapy is that imatinib kills CML cells but not CML primitive progenitors while IFNα preferentially target CML stem cells. Although IFNα was widely used to treat CML before the discovery of imatinib, the molecular mechanism of IFNα-mediated antileukemic effects is still unknown. It has been proposed that IFNα modulates gene expression, induces apoptosis, inhibits cell proliferation, and induces an immunomodulatory response [11]. Furthermore, high dose of interferon may lead to severe toxicity, including neurotoxicity and depression especially after 3–5 years of treatments [20]. Our results show that inhibition of EHMT1 and EHMT2 with chemical inhibitors enhances the type I interferon response. Thus the combination treatment of BIX01294 with interferon will significantly reduce the dosage of interferon required to achieve therapeutic level of the interferon response. This may reduce the side effects of the interferon. In addition, we demonstrate that inhibition of EHMT1 and EHMT2 not only augments the cytotoxicity of IFNα2a but also the cytotoxicity of imatinib. Therefore, inhibition of EHMT1 and EHMT2 will potentiate the efficacy of the imatinib and interferon combination therapy.

There are three phases of CML, chronic phase, accelerated phase and blastic phase. Interferon treatment is most effective in treating patient with chronic phase CML and less effective in treating accelerated and blastic phases CML [11]. Accelerated and blastic CML patients have poor prognosis and irresponsive to imatinib or interferon treatment. K562, BV173, KCL22 and KT1 cells were derived from blastic CML patients. Among the four cell lines, K562, KCL22 and BV173 are resistant to IFNα2a treatment (Figure 2A–C). Our findings show that BIX01294 treatment sensitizes the three interferon-resistant CML cell lines to IFNα2a. Moreover, we compared the type I interferon response between interferon resistant K562, KCL22 and BV173 cells and interferon sensitive KT1 cells. We demonstrated that KT1 cells have higher expression levels of several ISGs and are more resistant to viral infection than K562, KCL22 and BV173 cells. More importantly, ectopic expression of EHMT1 and EHMT2 in KT1 cells reduced the type I interferon response (Figure 7E). These results implying that the degree of type I interferon response of CML cells is regulated by EHMT1 and EHMT2. In addition, it has been shown that different level of H3K9 methylation at the promoters of ISGs contributes to the cell-type differences in ISGs expression [5]. In the study, the authors showed that the levels of H3K9 methylation inversely correlate with the scope and amplitude of ISGs expression in fibroblasts and dendritic cells. Consistently, we found that KT1 cells express EHMT1 and EHMT2 at slightly lower level compared to K562, BV173 and KCL22 cells. Further experiment will be required to determine if the levels of EHMT1, EHMT2 and H3K9 methylation at promoters of ISGs correlate with the responsiveness of CML patient to interferon treatment.

Our *in vitro* results demonstrated a new way to improve the efficacy of interferon against CML. It would be important to determine the effect of EHMT1 and EHMT2 inhibitors *in vivo*. However we were unable to extend our study with an *in vivo* animal study because both BIX01294 and UNC0638 have poor pharmacokinetic property *in vivo* [10]. We tested peritoneal, subcutaneous and retro-orbital injections of BIX01294 into mice, but found that less than 1% of total injected BIX01294 was detected in the blood (Raskatov J. and Ea C-K. unpublished results). In addition, a high dose of BIX01294 kills cells through an unknown mechanism [6] and our results show that a high dose of BIX01294 is toxic to all tested cell lines (Figure 2–3). Hence, further improvement of the pharmacokinetic properties of EHMT1 and EHMT2 specific chemical inhibitors is required.

Our results showed that inhibiting EHMT1 and EHMT2 with BIX01294 significantly reduced the H3K9me2 level at the promoters of *IFIT3*, *GBP3* and *β-globin* but not at the promoter of *GAPDH* (Figure 5A). Consistently, IFNα2a-induced expression of *IFIT3*, *GBP3* and *β-globin* (Figure 1 and data not shown) as well as the recruitment of RNA polymerase II to the promoters of these genes (Figure 5B) were enhanced in the cells treated with BIX01294. Our previous study showed that EHMT1 is dispensable for the H3K9me2 modification at the promoter of *β-globin* in HeLa cells [4]. However, in this study we found that BIX01294 treatment reduced the H3K9me2 level at the promoter of *β-globin* in K562 cells. Moreover, IFNα2a slightly induces the expression of *β-globin* in K562 cell but not in HeLa cells, and BIX01294 treatment enhances both the basal and IFNα2a-induced expression of *β-globin* in K562 cells but not in HeLa cells (data not shown). These results imply that EHMT1- and EHMT2-regulated gene may be cell type specific. The expression of *β-globin* is restricted to erythrocytes. K562 cells are of the erythroleukemia type. Thus *β-globin* is considered a permissive gene in K562 and a non-permissive gene in other cell types, such as HeLa cells, a cervical cancer cell line. EHMT1 and EHMT2 are mainly associated with euchromatin where most of the permissive genes are located, while SUV39H1 and SUV29H2 are mainly present in heterochromatin that is enriched with non-permissive genes [21], [22]. Further study to test if *β-globin* is located within euchromatin in K562 cells while within heterochromatin in other cell types, and thus regulates the accessibility of *β-globin* by EHMT1 and EHMT2 in different cell types, would provide valuable insight on the mechanism of EHMT1- and EHMT2-mediated gene regulation.

## Conclusion

Our results demonstrate that the inhibition of EHMT1 and EHMT2 with chemical inhibitors or RNAi sensitizes CML cells to interferon and imatinib treatments. We provide evidence that targeting EHMT1 and EHMT2 is a potential new approach to improve existing CML treatments, including imatinib and interferon therapies.

## Supporting Information

Figure S1 **BIX01294 and UNC0638 inhibit EHMT1 and EHMT2** ***in vivo*** **.** (**A**) K562 cells were incubated with 2.5 µM BIX01294 or 5 µM UNC0638 for twenty-four hours. Whole cell extracts were immunoblotted with the indicated antibodies (D: DMSO, B: BIX01294, U: UNC0638). (**B**) K562 cells were incubated with 2.5 µM BIX01294 for twenty-four hours followed by 1000 IU/ml IFNα2a stimulation for two hours. Whole cell extracts were immunoblotted with the indicated antibodies.
(TIF)

Figure S2 **Imatinib reduces the expression of BCR-ABL.** K562 cells were incubated with the indicated drugs for twenty-four hours. The expression of *BCR-ABL* was measured with RT-qPCR (**A**). Error bars represent the variation range of duplicate experiments. Whole cell extracts were immunoblotted with the indicated antibodies (**B**). IFN: IFNα2a, BIX: BIX01294, Im: Imatinib).
(TIF)

Figure S3 **Ectopic expression of FLAG-mEHMT1 and HA-mEHMT2.** (**A**) Whole cell extracts were prepared from KT1 empty vector or FLAG-mEHMT1-HA-mEHMT2 cells and analyzed by immunoblotting with FLAG-, HA- and PARP-specific antibodies. (**B**) Total RNAs were extracted from cells in (**A**) and the expression of *mEHMT1, mEHMT2* and *L32* were measured with RT-qPCR.
(TIF)

Table S1 **Sequence of qPCR primers.**
(DOCX)

Table S2 **Sequence of ChIP primers.**
(DOCX)
