# Supplementary figures and images for: Inhibition of Euchromatic Histone Methyltransferase 1 and 2 Sensitizes Chronic Myeloid Leukemia Cells to Interferon Treatment
Source: PLoS One. 2014 Jul 31;9(7):e103915. doi: 10.1371/journal.pone.0103915 (PMC4117596; doi:10.1371/journal.pone.0103915)

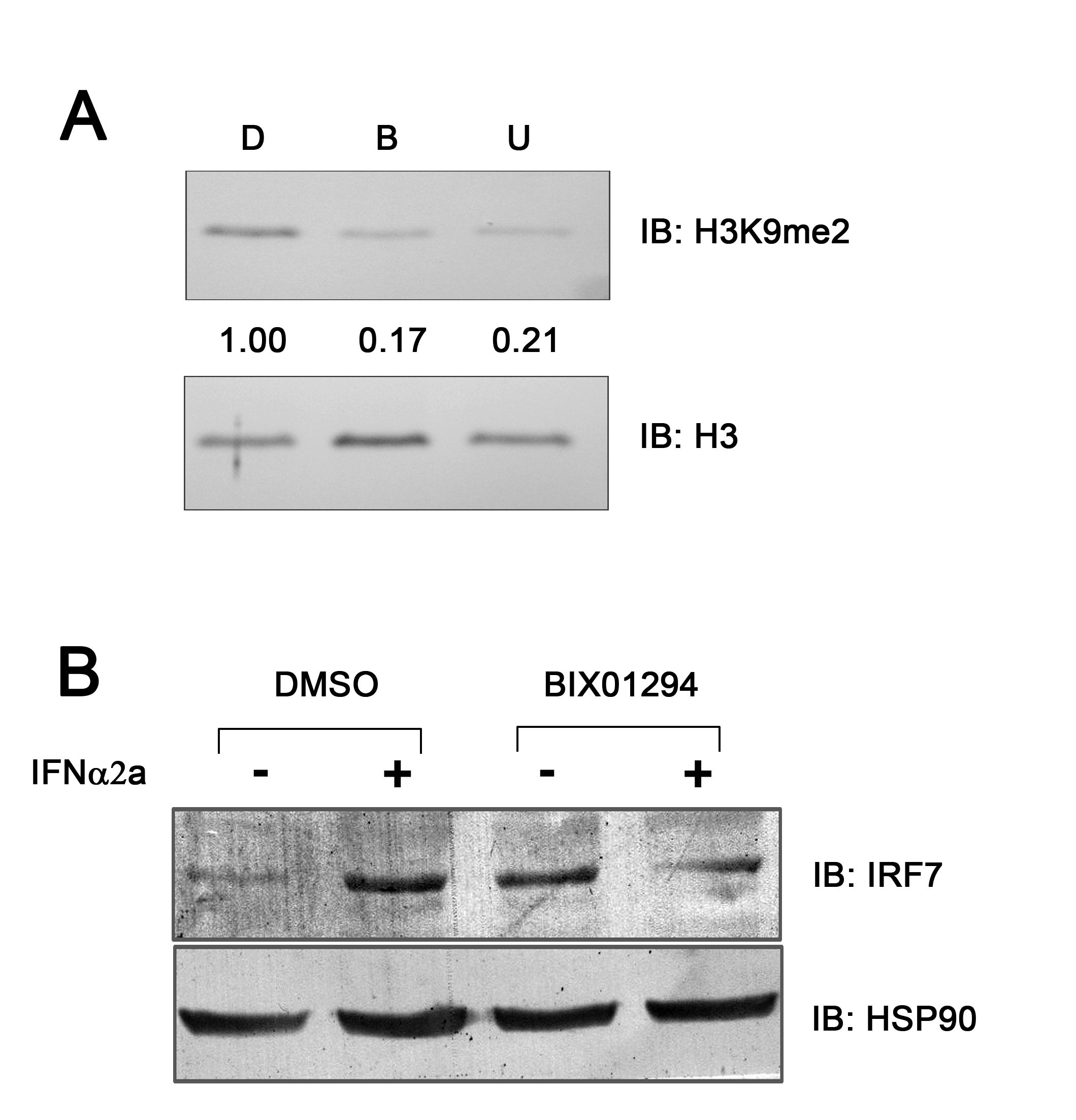

Supplement: Figure S1 — BIX01294 and UNC0638 inhibit EHMT1 and EHMT2 in vivo . (A) K562 cells were incubated with 2.5 µM BIX01294 or 5 µM UNC0638 for twenty-four hours. Whole cell extracts were immunoblotted with the indicated antibodies (D: DMSO, B: BIX01294, U: UNC0638). (B) K562 cells were incubated with 2.5 µM BIX01294 for twenty-four hours followed by 1000 IU/ml IFNα2a stimulation for two hours. Whole cell extracts were immunoblotted with the indicated antibodies. (TIF) [file pone.0103915.s001.tif]

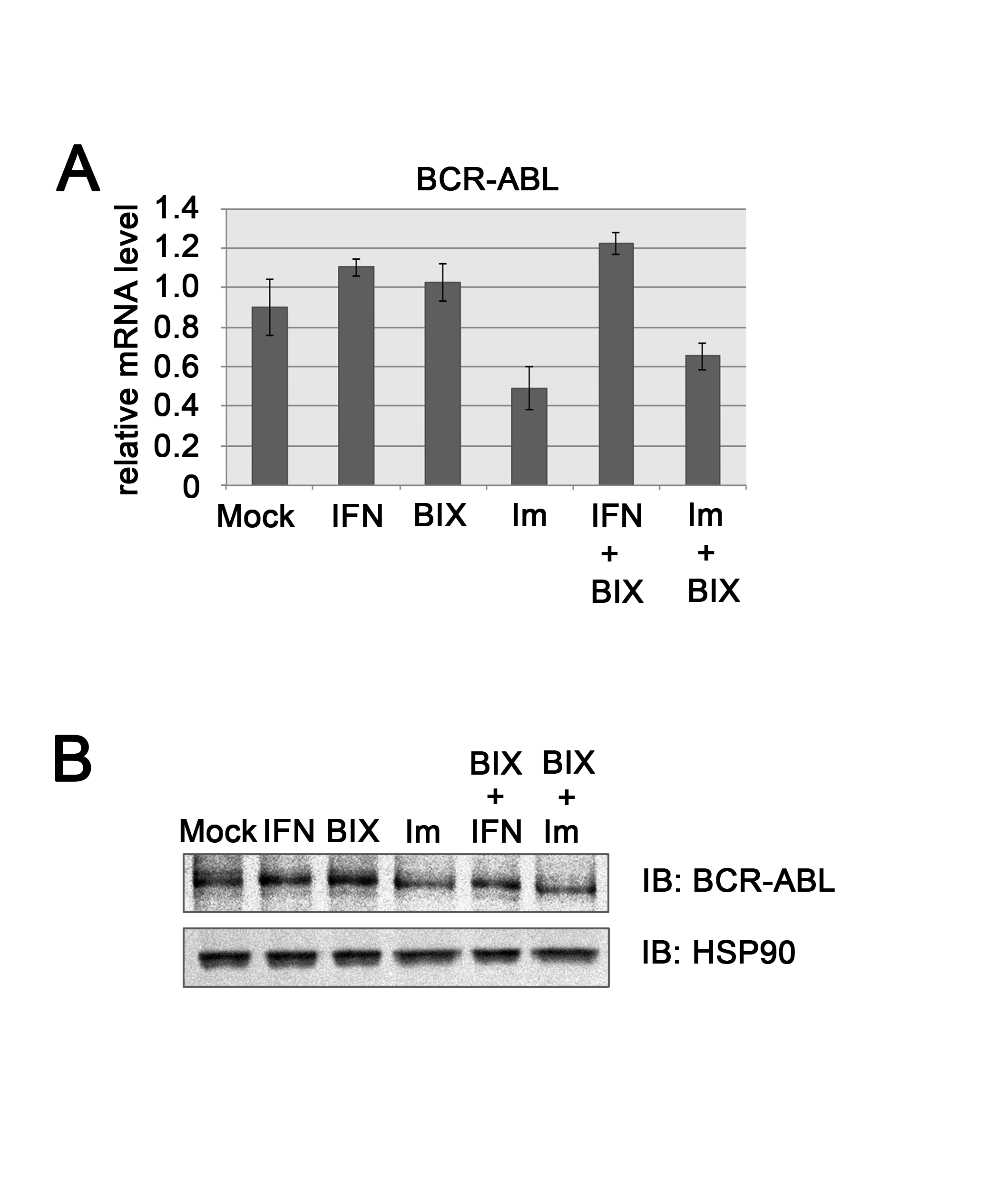

Supplement: Figure S2 — Imatinib reduces the expression of BCR-ABL. K562 cells were incubated with the indicated drugs for twenty-four hours. The expression of BCR-ABL was measured with RT-qPCR (A). Error bars represent the variation range of duplicate experiments. Whole cell extracts were immunoblotted with the indicated antibodies (B). IFN: IFNα2a, BIX: BIX01294, Im: Imatinib). (TIF) [file pone.0103915.s002.tif]

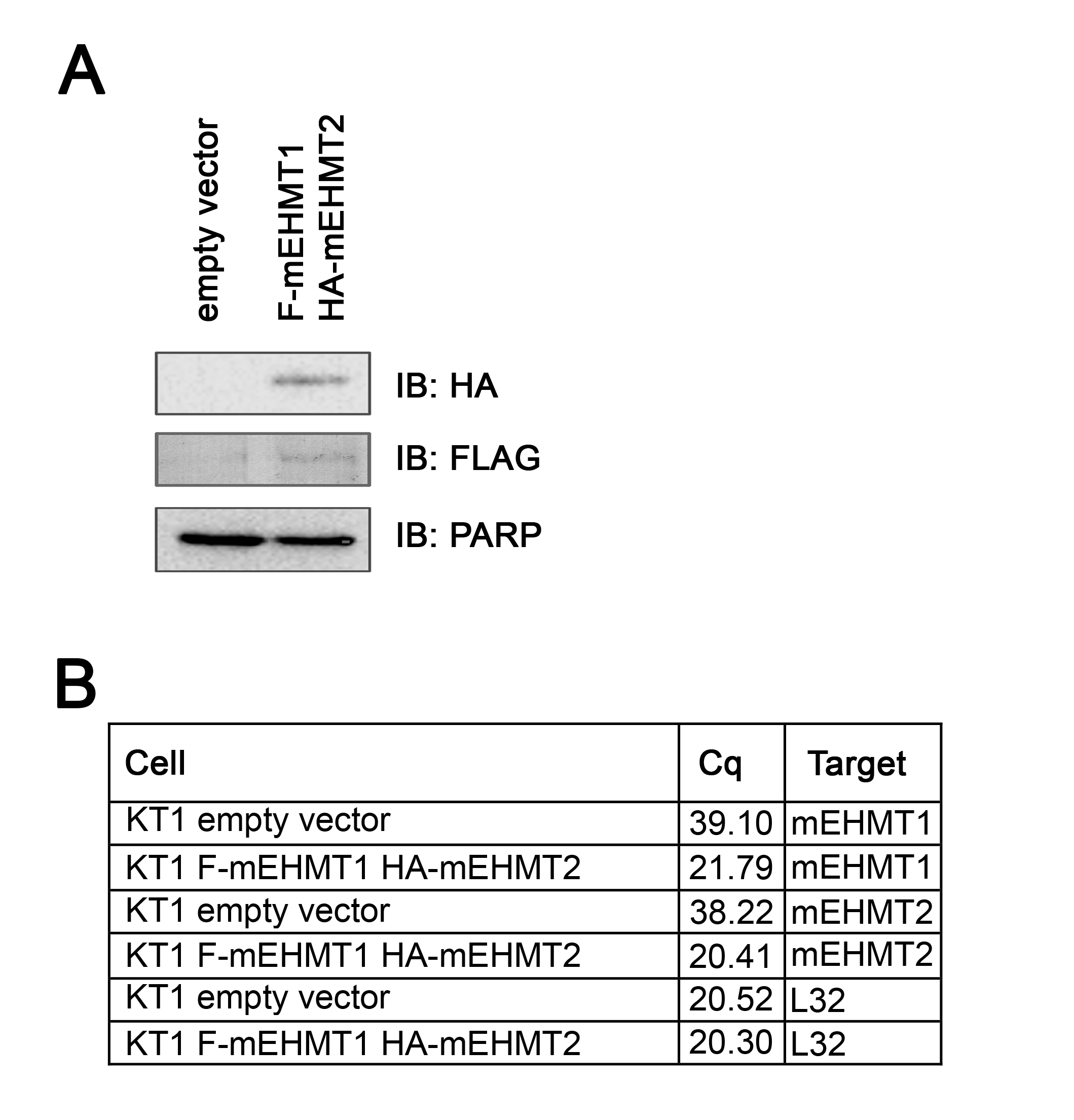

Supplement: Figure S3 — Ectopic expression of FLAG-mEHMT1 and HA-mEHMT2. (A) Whole cell extracts were prepared from KT1 empty vector or FLAG-mEHMT1-HA-mEHMT2 cells and analyzed by immunoblotting with FLAG-, HA- and PARP-specific antibodies. (B) Total RNAs were extracted from cells in (A) and the expression of mEHMT1, mEHMT2 and L32 were measured with RT-qPCR. (TIF) [file pone.0103915.s003.tif]
