# Supplementary material for: Inhibition of Euchromatic Histone Methyltransferase 1 and 2 Sensitizes Chronic Myeloid Leukemia Cells to Interferon Treatment
Source: PLoS One. 2014 Jul 31;9(7):e103915. doi: 10.1371/journal.pone.0103915 (PMC4117596; doi:10.1371/journal.pone.0103915)
Supplement: Table S1 — Sequence of qPCR primers. (DOCX) [file pone.0103915.s004.docx]

| hL32/5’ | AGCTCCCAAAAATAGACGCAC |
| --- | --- |
| hL32/3' | TTCATAGCAGTAGGCACAAAGG |
| hIFIT2/5' | GCGTGAAGAAGGTGAAGAGG |
| hIFIT2/3' | GCAGGTAGGCATTGTTTGGT |
| hIFIT3/5’ | GGGCAGACTCTCAGATGCTC |
| hIFIT3/3' | CAGTTGTGTCCACCCTTCCT |
| hOAS2/5' | AATGCCAGGAGAAGCTGTGT |
| hOAS2/3' | AAGATTACTGGCCTCGCTGA |
| hOAS3/5' | GTCAAACCCAAGCCACAAGT |
| hOAS3/3' | TGTAGGCACACCTGGTGGTA |
| hGBP1/5' | ACAAGCTGGCTGGAAAGAAA |
| hGBP1/3' | GTACACGAAGGTGCTGCTCA |
| hGBP3/5' | ACCAGGGGGCTATTGTCTCT |
| hGBP3/3' | CGGTCACAGACTCCTTGGAT |
| hEHMT1/5' | TACCAGCACTCTGGCCTCTT |
| hEHMT1/3' | GGAGCTTCCTGTCCTCTGTG |
| hb3a2/5' | GGGCTCTATGGGTTTCTGAATG |
| hb3a2/3' | CGCTGAAGGGCTTTTGAACT |
| hIRF7/5' | CTTCGTGATGCTGCGAGATA |
| hIRF7/3' | TTGGAGTCCAGCATGTGTGT |
| mEHMT1/5’ | AGGCCACCATCACCTACAAG |
| mEHMT1/3’ | AAAATGAGTGGTGGGTCAGC |
| mEHMT2/5’ | CTCAGGGCCTTCACTGACTC |
| mEHMT2/3’ | GATGACCAAGGCTTTTTCCA |

**Table S1: Sequence of qPCR primers.**
