# Supplementary material for: Inhibition of Euchromatic Histone Methyltransferase 1 and 2 Sensitizes Chronic Myeloid Leukemia Cells to Interferon Treatment
Source: PLoS One. 2014 Jul 31;9(7):e103915. doi: 10.1371/journal.pone.0103915 (PMC4117596; doi:10.1371/journal.pone.0103915)
Supplement: Table S2 — Sequence of ChIP primers. (DOCX) [file pone.0103915.s005.docx]

**Table S2: Sequence of ChIP primers.**

| hHBB/pro/5' | TGG TAT GGG GCC AAG AGA TA |
| --- | --- |
| hHBB/pro/3' | GCT CCA CAG GGT GAG GTC TA |
| hIFIT3/pro2/5’ | ATG AGG CAT ATC CAG CTT CC |
| hIFIT3/pro2/3’ | AAG GCC ATA CTC ACC AAC CA |
| hGBP3/pro/5’ | CAA TTC TCA AAC AGA TCT CCA ATC |
| hGBP3/pro/3’ | TGC AAT TTC TGG ATT CTG CT |
| hGAPDH/pro/5' | TAC TAG CGG TTT TAC GGG CG |
| hGAPDH/pro/3' | TCG AAC AGG AGG AGC AGA GAG CGA |
